# Downregulation of hsa-miR-30b-3p Inhibits the Oncogenicity of Lung Adenocarcinoma by Targeting the *METTL7B* Gene

**DOI:** 10.1155/2022/6883140

**Published:** 2022-05-20

**Authors:** Ning Li, Rui Li, Bin Song, Hong Ge, Yiqing Qu

**Affiliations:** ^1^Department of Pulmonary and Critical Care Medicine, Qilu Hospital, Cheeloo College of Medicine, Shandong University, Shandong Key Laboratory of Infectious Respiratory Diseases, Jinan 250012, China; ^2^Department of Pulmonary and Critical Care Medicine Zibo Central Hospital, Zibo 255020, China

## Abstract

**Objective:**

Lung adenocarcinoma (LUAD) is one of the malignant tumors with the highest morbidity and mortality rates worldwide. Although surgery-based combination therapy can greatly improve the prognosis of LUAD patients, the overall outcome is still poor, and there is an urgent need to develop new and effective treatment alternatives for LUAD. The microRNA (miRNA) miR-30b-3p is a typical multifunctional miRNA that has been reported to promote the development of various malignancies. In this study, we investigated the effects of miR-30b-3p on the biological properties of LUAD and the possible mechanisms involved to provide new ideas for the development of more effective treatment options for LUAD.

**Methods:**

Fluorescence quantitative real-time polymerase chain reaction (qRT-PCR) was used to measure the expression of miR-30b-3p and its target *METTL7B* gene in tumor tissues and adjacent noncancerous lung tissues of LUAD patients and to determine their correlation. The effect of miR-30b-3p on the biological properties of LUAD was investigated, after transfection of miR-30b-3p mimics or scramble miRNA (negative control) in LUAD cells, using various approaches, including by measuring cell proliferation using CCK-8 and Edu assays, cell invasion by Transwell assay, and apoptosis and cell cycle distribution by flow cytometry. Additionally, rescue assays were performed to verify the effect of METTL7B on miR-30b-3p function.

**Results:**

In LUAD patients, low expression of miR-30b-3p and high expression of *METTL7B* in tumor tissues were significantly and negatively correlated with those in adjacent noncancerous lung tissues of the LUAD patients. It was also found that miR-30b-3p inhibits the proliferation and invasion of LUAD cells, promotes apoptosis, and changes the cell cycle distribution. *METTL7B* is a target gene of miR-30b-3p. METTL7B overexpression significantly reversed the biological effects of miR-30b-3p overexpression, including inhibition of cell proliferation and invasion, promotion of apoptosis, and alteration of the cell cycle distribution.

**Conclusions:**

The miR-30b-3p alters the biology of LUAD by negatively regulating *METTL7B* expression, indicating the importance of the miR-30b-3p/METTL7B pathway in the development of LUAD.

## 1. Introduction

Lung cancer is the second in incidence and the top leading cause of cancer death worldwide [1]. The five-year survival rate for lung cancer is extremely low, with a survival rate of 19% according to Cancer statistics 2020, which is only higher than pancreatic cancer at 9% and liver cancer at 18% [2]. Also, due to its high incidence, lung cancer ranks first in the number of deaths from cancer-related deaths each year. Lung cancer is highly prone to metastasize to lymph nodes and through the bloodstream, with 57% of all initial diagnoses being metastatic lung cancer, and a five-year survival rate of only 5% for this group of patients [3, 4]. Therefore, early diagnosis and treatment are extremely important for improving patient prognosis.

Lung adenocarcinoma (LUAD) accounts for approximately 55% of all non-small-cell lung cancer cases [5, 6]. Due to its insidious early symptoms, most patients with LUAD are diagnosed at an advanced stage. The five-year survival rate for LUAD has been reported to be less than 17% [6]. LUAD is the most prevalent type of cancer among nonsmokers and women. The current common treatment is surgical resection combined with radiation therapy and drug therapy [7, 8]. Although significant efficacy has been achieved with EGFR receptor tyrosine kinase inhibitors and immune checkpoint inhibitors in patients with EGFR mutation-positive and high PDL1-expressing LUAD, patients with LUAD still face a high risk of death due to the high propensity of lung cancer to postoperative recurrence and development of drug resistance [9]. Therefore, it is important to study the molecular mechanisms of LUAD development in order to identify earlier tumor markers and drug targets to improve clinical diagnosis and treatment.

MicroRNAs (miRNAs) are a class of endogenous evolutionarily conserved noncoding small RNA molecules with a length of about 18-22 nucleotides [10, 11]. The processing of miRNAs can be divided into two steps. The first step is the enzymatic cleavage of the long polycistronic (about 300-1,000 bases in length) primary miRNA transcript (pri-miRNA) in the nucleus by the Drosha/DGCR8 complex, into a miRNA precursor (pre-miRNA) of about 70-90 bases in length. In the second step, the pre-miRNA is translocated from the nucleus to the cytoplasm and cleaved by the Dicer enzyme into a double-stranded RNA molecule of about 20-24 bp [12]. One strand is quickly degraded and the other strand becomes the mature miRNA that is involved in gene expression regulation. The strand contains a phosphate group at the 5′ end and a hydroxyl group at the 3′ end, whereas functional RNAs and most oligonucleotide degradation fragments do not have these features, allowing miRNAs to be distinguished [13]. As major regulators of transcribed genes at the posttranscriptional level, miRNAs bind the 3′-untranslated region (3′-UTR) of target gene mRNAs through complementary pairing and regulate more than 1/3 of human target genes [14, 15]. At least 2,000 miRNAs have been identified in humans, which are classified into protooncogenic miRNAs and oncogenic miRNAs according to their expression levels in tumors [16]. In addition, miRNAs can regulate downstream protooncogenes and oncogenes. Once miRNAs are abnormally expressed, they can lead to disruption of several functional processes in tumor cells, including cell proliferation, migration, invasion, drug resistance, and tumor recurrence [17]. Therefore, miRNAs play an important role in tumor development and can be used as potential tumor markers and targets.

Studies have found that miRNAs are inextricably linked to the development and progression of LUAD [18, 19]. For instance, on the one hand, when the expression of a miRNA is upregulated in LUAD, it can play a prooncogenic role in the development of LUAD by suppressing the expression of its target oncogenes. On the other hand, when the expression of a miRNA is downregulated in LUAD, it can play an antioncogenic role in the development of LUAD by promoting the expression of its target oncogenes [20, 21]. Notably, miR-30 family is an important member of miRNA family containing 6 mature miRNA molecules (miR-30a, miR-30b, miR-30c-1, miR-30c-2, miR-30d, and miR-30e). As a miRNA, miR-30b-3p has been reported to be tightly associated with tumor development [22]. In glioma, miR-30b-3p was found to be closely associated with metastasis by upregulating RECK [23]. However, the function of miR-30b-3p in LUAD and its mechanism of action have not been fully investigated. Also, METTL7B, a member of the methyltransferase-like protein family comprising more than 27 members [24], is a recognized tumor target that has received considerable attention in recent years, but its function has not been fully elucidated. There is growing evidence that METTL7B plays an important role in the progression of malignancies, including primary thyroid cancer, breast cancer, and non-small-cell lung cancer [25].

## 2. Materials and Methods

### 2.1. Tissue Specimens

The tissues were collected from patients with LUAD undergoing radical or palliative resection in the Department of Thoracic Surgery at Qilu Hospital of Shandong University from May 2019 to September 2020. The LUAD tissues were derived from surgical or bronchoscopic biopsy of lung tumor and normal lung tissue adjacent to the tumor. The' tumor specimens of the patients were pathologically confirmed. None of the patients receive radiotherapy or chemotherapy prior to surgery, and specimen collection was approved by the Ethics Committee of Qilu Hospital of Shandong University (Jinan, China). All participating patients were informed of the study procedure and associated risks and signed an informed consent form. All fresh samples were immediately stored in liquid nitrogen. Inclusion criteria included (1) informed consent signed by the patient or family and reported to the ethics committee of the Qilu Hospital of Shandong University, (2) clear pathological diagnosis, (3) primary lung adenocarcinoma, and (4) complete clinical history information. Exclusion criteria included (1) having received radiotherapy, chemotherapy, tumor immunotherapy and other tumor-specific treatments, (2) comorbid immune system diseases, (3) comorbid chronic wasting diseases and infectious diseases, and (4) comorbid pregnancy or other types of malignancies.

### 2.2. Cell Recovery and Passaging

The LUAD cell lines, A549, PC-9, and H1975, and the human bronchial epithelial cell line BEAS-2B were removed from liquid nitrogen and rapidly thawed in a water bath at 37°C with gentle shaking. Then, the medium containing the thawed cells was transferred to a 15 mL centrifuge tube, further diluted with complete medium, and centrifuged at 1,000 rpm for 3 min. After discarding the supernatant, the cells were resuspended in complete medium, and the cell suspension was transferred to a cell culture flask and incubated in a cell culture incubator at 37°C with 5% CO_2_. The cells were passaged when they reached 80-90% confluence, by aspirating the medium and washing the cells 3 times with a phosphate-buffered saline (PBS) (Beyotime Bio Inc., Shanghai, China) solution. Then, the cells were detached by trypsin (Beyotime Bio Inc., Shanghai, China) treatment, resuspended in complete medium, and transferred to a centrifuge tube and centrifuged. After centrifugation, the supernatant was discarded and the precipitated cells were resuspended in complete medium and transferred into a new culture flask and incubated in an incubator at 37°C, with 5% CO_2_.

### 2.3. Transfection

The day before transfection, when the cells in the cell culture flasks are grown to log phase, trypsin digestion is centrifuged and counted with medium dilution and inoculated in culture dishes (at a density of approximately 4 × 10^5^/dish) so that the cells can reach 60%-70% cell density at the time of transfection, and cell spreads are made using normal medium containing serum, without antibiotics. On the day of transfection, mimics as well as the corresponding controls were removed from the -20°C refrigerator. Depending on the final concentration, a certain number of mimics with a starting concentration of 20 *μ*M was aspirated and diluted to the transfection working concentration of mimics 50 nM using opti-MEM (Solarbio Bio Inc., Beijing, China) serum-free medium, and 500 *μ*L of liquid was prepared and mixed by repeated gentle blowing. Aspirate 490 *μ*L opti-MEM serum-free medium to dilute 10 *μ*L liposome 2000 transfection reagent, mix gently, and leave for 5 min at room temperature. Mix the two mixtures of diluted RNA and diluted Liposome 2000 transfection reagent together (total volume 1 mL), blow gently, and keep at room temperature for 20 min. Remove the cells laid out the day before transfection, remove the old culture medium and replace it with 3 mL opti-MEM serum-free medium, add the above transfection mixture directly to the dish, and shake it slowly to mix it well. Incubate in 37°C, 5% CO_2_ incubator for 4-6 h. Discard the old culture medium, replace with fresh normal medium containing serum, and continue to incubate in 37°C cell incubator for 48 h for the next experiment.

### 2.4. Quantitative Real-Time Polymerase Chain Reaction (qRT-PCR)

Total RNA was extracted using the TRIzol method. For RNA extraction from tissues, about 50 mg of freshly frozen tissue specimens from lung cancer patients was transferred to separate ice-cold glass homogenizers, containing 1 mL of RNAiso Plus reagent (Takara Bio Inc., Kusatsu, Japan) solution, and ground until tissue cells were fully lysed. For RNA extraction from cell lines, after growing the cells to confluence in 6-well plates, the cells were rinsed twice with PBS and then fully lysed by adding 1 mL of RNAiso Plus reagent solution into each well. Subsequently, after transferring tissue and cell lysates to separate 1.5 mL Eppendorf tubes and adding 200 *μ*L of chloroform to teach tube, the lysate-chloroform mixtures were thoroughly mixed, allowed to stand on ice for 3 min, and centrifuged at 12,000 g/min, 4°C for 15 min. Afterward, total RNA was precipitated with isopropyl alcohol and then washed with 75% ethanol. After evaluating the quality and integrity of the extracted total RNA, it was used to perform fluorescence quantitative real-time polymerase chain reaction (qRT-PCR) on a Roche LightCycler 480 Fluorescence Real-Time PCR System (Roche Diagnostics AG, Rotkreuz, Switzerland).

### 2.5. Western Blot Analysis

Total protein was extracted from cells cultured in six-well plates. After washing the cells in each well with cold PBS, the cells were lysed by adding 200 *μ*L of RIPA lysis buffer (Solarbio Bio Inc., Beijing, China) to each well, mixing and incubating on ice for 30 min. Afterwards, each protein lysate was transferred to a 1.5 mL Eppendorf tube and centrifuged at 12,000 rpm/min at 4°C for 10 min, and the supernatant was dispensed into 1.5 mL Eppendorf tubes and stored at -80°C until analyzed. Subsequently, after solubilizing the protein on ice, the appropriate amount of protein was mixed with 4x protein loading buffer and fully denatured by placing in boiling water for 5 min. Then, the corresponding loading volume, containing 30 *μ*g/sample, was slowly loaded into the sodium dodecyl sulfate-polyacrylamide gel electrophoresis (SDS-PAGE) gel. Electrophoretically separated proteins were electrotransferred onto polyvinylidene difluoride (PVDF) membranes (Millipore Bio Inc., Massachusetts, United States), which were blocked with 5% skim milk powder solution. After washing the membranes with tris-buffered saline-Tween 20 (TBST) buffer solution, each membrane was incubated overnight at 4°C with the corresponding primary antibody METTL7B (Proteintech Group, Inc., Rosemont, USA) (dilution ratio 1 : 2000). Afterwards, the membranes were washed 4 times with TBST solution for 5 min and then incubated with the secondary antibody for 1 h, at room temperature. Immunoreactive protein bands were visualized by the enhanced chemiluminescence (ECL) detection method, followed by image acquisition and quantification of the immunoreactive protein bands by densitometry.

### 2.6. Measurement of Apoptosis and Cell Cycle Distribution by Flow Cytometry

After harvesting by trypsinization, the cells were double stained with fluorescein isothiocyanate- (FITC-) Annexin V and propidium iodide (PI) using the FITC-Annexin V Apoptosis Detection Kit (BD Biosciences, San Jose, CA, USA) and analyzed by flow cytometry to measure apoptosis. Also, following trypsinization, the cells were stained with propidium iodide using the CycleTEST™ PLUS DNA Reagent Kit (BD Biosciences) and analyzed by flow cytometry to measure the cell cycle distribution using the FACScan Cell Quest™ software (BD Biosciences).

### 2.7. Transwell Assay

Cell invasion assays were measured by Transwell chamber (8 *μ*m pore size, Corning); the Transwell chambers were also Matrigel-coated. The lower chamber was filled with 500 *μ*L of 20% FBS medium. Transfected LUAD cells (6 × 10^4^) in 200 *μ*L of serum-free medium were gently loaded onto each filter insert (upper chamber) and then incubated at 37°C for 48 h. The migratory cells were stained blue, visualized under and inverted microscope, and then counted.

### 2.8. Establishment of Subcutaneously Implanted Tumors in Nude Mice

BALB/c nude mice were selected for subcutaneous tumor implantation experiments due to their lack of a thymus and lack of immune response. BALB/c, 4-week old, 13-15 g, homozygous SPF (specific pathogen-free) grade female nude mice were used in this study. The mice were housed in an animal room maintained at a constant temperature of 25 ± 2°C and constant humidity of 45-50%, with a 12 : 12 h light and dark cycle. The feed, bedding, and drinking water were autoclaved (45 min, 120°C). The water for nude mice was acidified with hydrochloric acid to a pH 2.5-3.0. The mice were housed with 5 mice in each cage, and the cages were cleaned and the bedding was changed 1-2 times a week. The LUAD A549 cell line was cultured to logarithmic growth stage, routinely trypsinized, collected by centrifugation at 5,000 rpm for 5 min, and washed 3 times with cold PBS. After centrifugation, the cells were resuspended in PBS. The resuspended cells were mixed with stromal gel at a ratio of 5 : 1 by volume to prepare a cell suspension, which was used as the inoculum suspension for individual nude mice. A volume of 120 *μ*L of the cell suspension was subcutaneously injected into the flanks of ten 4–6-week-old female nude mice, which were randomly divided into the miR-30b-3p overexpression group and the control group. The survival status of the nude mice was observed daily. Tumors were generated on the posterior flank of the nude mice, and injections of miR-30b-3p Agomir and NC were started when tumors reached a size of 5 mm × 5 mm; the injections were repeated every three days for 2 to 4 weeks. The experiment was terminated after 3 weeks by euthanasia of the tumor-bearing nude mice. The subcutaneous tumors of the nude mice were removed and t weighed.

### 2.9. Statistical Analysis

The statistical software SPSS version 20.0 (IBM Corporation, Armonk, NY, USA) was used for statistical analysis. The results were expressed as the mean + standard deviation, and the *t*-test was used to compare the difference in means between groups, and *P* < 0.05 was considered to be statistically significant difference.

## 3. Results

### 3.1. Low Expression of miR-30b-3p in LUAD Tissues and Cell Lines

**Figure 1:**
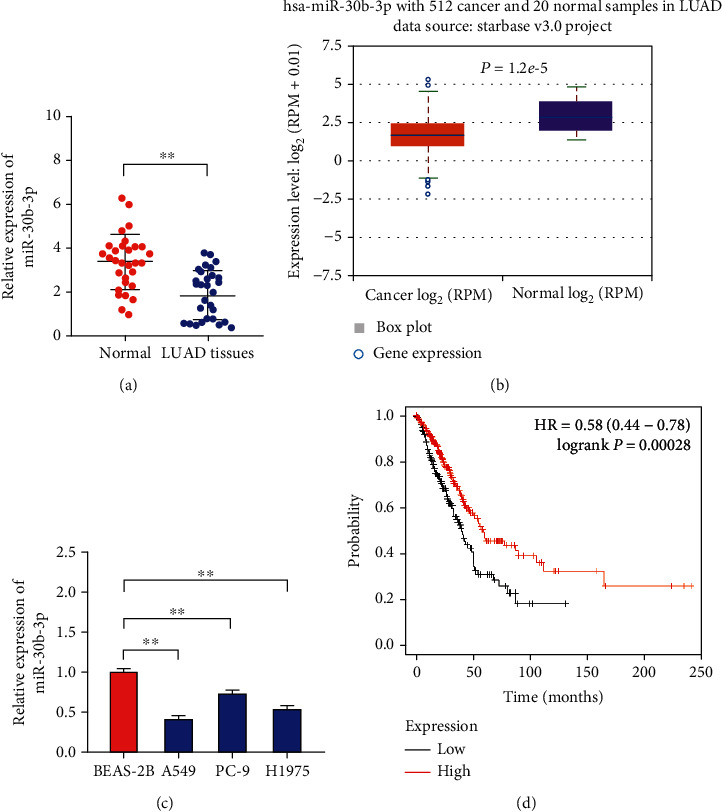
Low expression of miR-30b-3p in LUAD tissues and cell lines. (a) qRT-PCR analysis of miR-30b-3p expression in LUAD and paracancerous tissues. (b) The ENCORI platform was used to determine the expression of miR-30b-3p LUAD tissues in TCGA database. *Note*: this image was obtained straight from the ENCORI database. (c) qRT-PCR analysis of miR-30b-3p expression in the LUAD cell lines A549, PC-9, and H1975 and the human bronchial epithelial cell line BEAS-2B. (d) Using the online Kaplan-Meier Plotter database, we examined the relationship between miR-30b-3p expression and overall survival time (OS) in patients with LUAD. Note: this image was directly obtained from the Kaplan-Meier Plotter. ^∗∗^*P* < 0.01.

We analyzed LUAD tissues and paracancerous tissues by qRT-PCR analysis to identify the differential expression levels of miR-30b-3p in these tissues. The analysis revealed that the expression of miR-30b-3p was much lower in LUAD tissues than that in normal lung tissues (Figure 1(a)). Additionally, we also examined the expression of miR-30b-3p in a large sample of LUAD tissues from The Cancer Genome Atlas (TCGA) database through the ENCORI platform (https://starbase.sysu.edu.cn/) [26]. Our findings demonstrated consistent low expression of miR-30b-3p in LUAD tissues (Figure 1(b)). We further verified the expression level of miR-30b-3p in LUAD cells by qRT-PCR analysis, and the results revealed that miR-30b-3p was similarly expressed at much lower levels in the LUAD cell lines A549, PC-9, and H1975 than in the human bronchial epithelial cell line BEAS-2B (Figure 1(c)). We also examined the association between miR-30b-3p expression and overall survival time (OS) of LUAD patients using the online Kaplan-Meier Plotter database [27] and found that patients with low miR-30b-3p expression had a worse OS (HR = 0.58, 95% CI: 0.44-0.78, log-rank *P* = 0.00028, Figure 1(d)).

### 3.2. The miR-30b-3p Inhibits the Proliferation and Invasion Ability of LUAD Cells, Promotes Apoptosis, and Changes the Cell Cycle Distribution

**Figure 2:**
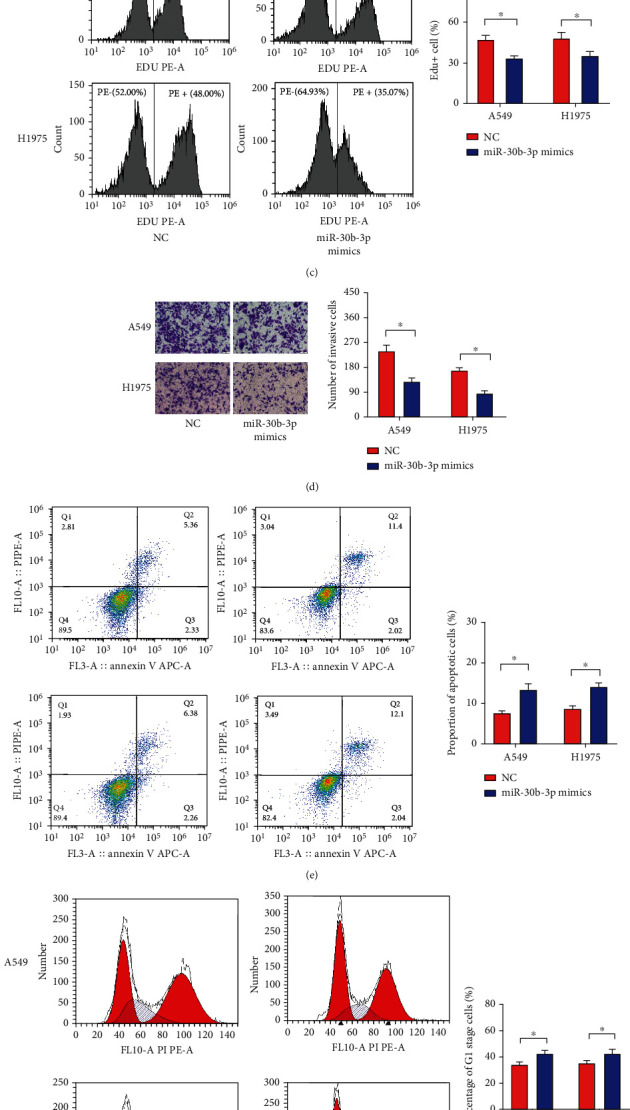
miR-30b-3p expression affects the biological function of LUAD cells. (a) qRT-PCR analysis of miR-30b-3p expression in LUAD cells. (b) Measurement of the OD value of LUAD cells by CCK-8 assay. (c) Detection of LUAD cell proliferation by the EdU incorporation assay. (d) Analysis of LUAD cell invasion by the Transwell assay. (e) Measurement of LUAD cell apoptosis by flow cytometric assay. (f) Analysis of cell cycle distribution of LUAD cells by flow cytometry. ^∗^*P* < 0.05.

To examine the biological role of miR-30b-3p in LUAD, we transfected A549 and H1975 cells with miR-30b-3p mimics. Two LUAD cell lines transfected with miR-30b-3p mimics showed a substantial increase (up to 140-fold) in miR-30b-3p expression (Figure 2(a)). The proliferation differences between the LUAD cell lines overexpressing miR-30b-3p and NC were detected using the cell-counting kit 8 (CCK-8) and EdU incorporation assays. Overexpression of miR-30b-3p resulted in a decrease in the proliferative capacity of cells (Figures 2(b) and 2(c)). Subsequently, we examined the effect of miR-30b-3p on the invasive ability of LUAD cells using the Transwell assay. The results revealed that the number of invasive cells in the miR-30b-3p mimics group was significantly lower than that in the NC group in A549 and H1975 cells (Figure 2(d)), indicating that miR-30b-3p inhibited the invasive ability of LUAD cells. We also investigated whether miR-30b-3p promotes apoptosis using flow cytometric analysis to compare the fraction of apoptotic cells in the miR-30b-3p mimic group to that in the NC group (Figure 2(e)). The results revealed that the G1 phase was prolonged in both A549 and H1975 cells overexpressing miR-30b-3p, with no significant alterations observed in the S or G2/M phases (Figure 2(f)).

### 3.3. miR-30b-3p Targets the *METTL7B* Gene

**Figure 3:**
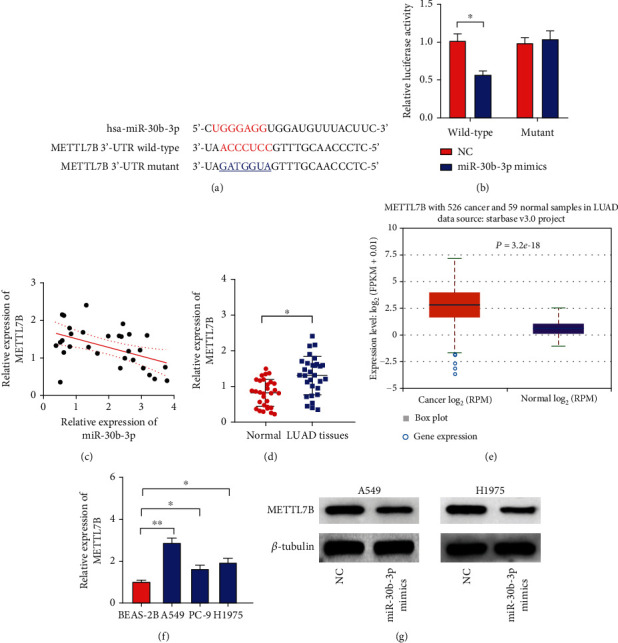
miR-30b-3p targets the *METTL7B* gene. (a) Prediction of *METTL7B* as a target of miR-30b-3p by bioinformatics analysis. (b) Validation of the targeting of *METTL7B* by miR-30b-3p by a dual luciferase assay. (c) Expression and correlation of miR-30b-3p and *METTL7B* in LUAD tissues were determined and analyzed by qRT-PCR. (d) qRT-PCR analysis was used to determine the expression level of *METTL7B* in 30 LUAD tissues and normal lung tissues. (e) ENCORI platform-based analysis of *METTL7B* expression in a large sample of LUAD tissues. (f) qRT-PCR analysis of *METTL7* expression in the LUAD cell lines A549, PC-9, and H1975 and the human bronchial epithelial cell line BEAS-2B. (g) Detection of METTL7 protein expression in miR-30b-3p-overexpressing LUAN cells by Western blot analysis. ^∗^*P* < 0.05.

We found that *METTL7B* might be a target gene of miR-30b-3p (Figure 3(a)) [28–30]. To determine whether *METTL7B* is a miR-30b-3p downstream target gene, we used a series of vectors constructed to perform luciferase assays. Transfection of wild-type or mutant *METTL7B* 3′-UTR region vectors into miR-30b-3p overexpressing cells or NC cells was performed to evaluate the effect miR-30b-3p overexpression on luciferase activity. The results indicated that the miR-30b-3p overexpression group showed significantly lower luciferase activity than the NC group in the wild-type *METTL7B* 3′-UTR region vector-transfected cells, but there was no significant difference in luciferase activity between the miR-30b-3p overexpression and NC groups in the mutant *METTL7B* 3′-UTR region vector-transfected cells. This finding indicates that miR-30b-3p may bind to the 3′-UTR of *METTL7B* and regulate its expression (Figure 3(b)). The expression analysis by qRT-PCR was used to determine the expression levels of miR-30b-3p and *METTL7B* gene in tissue specimens surgically harvested from 30 LUAD patients and to examine the association between them. The findings revealed a negative correlation between miR-30b-3p levels in LUAD tissues and *METTL7B* mRNA expression (*r* = −0.4806; *P* < 0.01; Figure 3(c)). In addition, we also examined *METTL7B* expression in LUAD by qRT-PCR analysis to determine the expression level of *METTL7B* in the 30 LUAD tissues and normal lung tissues. The results showed that the expression of *METTL7B* was significantly higher in LUAD tissues than in normal lung tissues (Figure 3(d)). Furthermore, we used the ENCORI platform to determine the expression of *METTL7B* in LUAD tissues from TCGA database. The findings revealed that *METTL7B* expression was significantly increased in LUAD tissues (Figure 3(e)). Similarly, *METTL7B* expression was considerably higher in the three LUAD cell lines than in the BEAS-2B cells (Figure 3(f)). Moreover, we also performed a Western blot analysis of METTL7B expression in LUAD cell lines overexpressing miR-30b-3p and found that METTL7B protein expression was decreased in A549 and H1975 cell lines overexpressing miR-30b-3p compared to the NC group (Figure 3(g)).

### 3.4. Tumor Formation in Nude Mice to Corroborate the Oncogenic Effect of miR-30b-3p *In Vivo*

**Figure 4:**
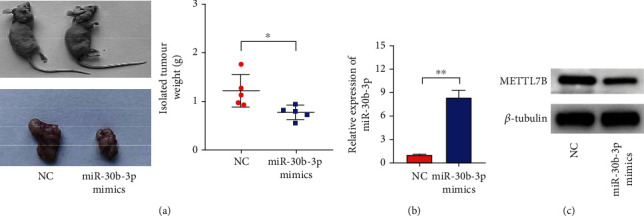
miR-30b-3p affects the tumorigenicity of LUAD cells in nude mice. (a) Weight analysis of tumorigenic tumors of different groups of LUAD cells in nude mice. (b) Measurement of miR-30b-3p expression in isolated tumors by qRT-PCR analysis. (c) Measurement of METTL7B expression in isolated tumors by Western blot analysis. ^∗^*P* < 0.05.

We established a LUAD cell subcutaneous implantation tumor model in nude mice based on the A549 cell line transfected with miR-30b-3p or NC. After seven injections of the miR-30b-3p Agomir and NC, i.e., day 21 after inoculation, the nude mice were sacrificed by decapitation. The tumors of the mice were dissected, weighed, and photographed. The results showed that the morphology of the implanted tumors was solid mass-like, partly lobulated, and most of them were clearly defined from the surrounding tissues, with no obvious necrosis was observed. Few of them had infiltration to the surrounding skin and tissues, and there were obvious differences in the size of tumors between the two groups. The weight of the tumors from the two groups revealed that the tumors from the miR-30b-3p overexpression group were smaller than those from the NC group (Figure 4(a)). The qRT-PCR analysis was performed on the isolated tumor tissues to measure miR-30b-3p expression, and, as anticipated, the results showed that the miR-30b-3p overexpression group had higher miR-30b-3p expression levels than the negative control group (Figure 4(b)). Additionally, we measured *METTL7B* expression by Western blot analysis and qRT-PCR, and the results showed that the expression of METTL7B was reduced in the miR-30b-3p overexpression group compared to the NC group (Figure 4(c)).

### 3.5. Rescue Experiments to Corroborate That METTL7B Reverses the Biological Role of miR-30b-3p in LUAD Cells

**Figure 5:**
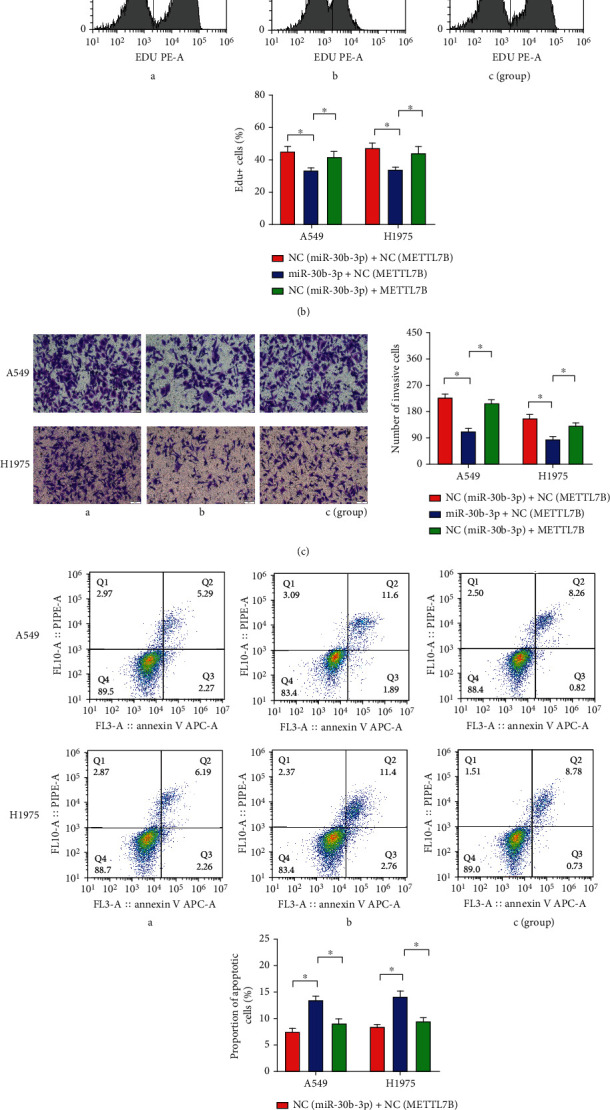
METTL7B reverses the biological role of miR-30b-3p in LUAD cells. (a) Measurement of OD value of LUAD cells by the CCK-8 assay. (b) Measurement of LUAD cell proliferation by EdU incorporation assay. (c) Detection of LUAD cell invasion by Transwell assay. (d) Measurement of LUAD cell apoptosis by flow cytometric analysis. (e) Detection of LUAD cell cycle distribution by flow cytometry. ^∗^*P* < 0.05.

To determine whether miR-30b-3p-mediated inhibition of METTL7B expression is the main mechanism for inhibiting LUAD development and progression, vectors containing only the core coding sequence of *METTL7B* without the 3′-UTR and control vectors were separately transfected into miR-30b-3p overexpressing cells. In addition, proliferation, invasion, apoptosis, and cycle assays were performed to determine whether restoration of METTL7B expression could reverse the effect of miR-30b-3p overexpression on LUAD cells. The results revealed that METTL7B overexpression significantly reversed the biological effects of miR-30b-3p overexpression, including inhibition of proliferation (Figures 5(a) and 5(b)) and invasion (Figure 5(c)), promotion of apoptosis (Figure 5(d)), and change of the cell cycle distribution (Figure 5(e)). These findings suggest that METTL7B plays a prooncogenic role and the miR-30b-3p/METTL7B axis is involved in the process of LUAD development and progression.

## 4. Discussion

A miRNA is a small noncoding RNA molecule that precisely regulates the physiological activity of cells and interferes with the translation of target gene proteins by promoting the degradation of their mRNAs. A turning point in the history of miRNA research occurred when the role of miRNAs in cancer development and progression was discovered, and significant progress was made in understanding the role of miRNAs in the pathogenesis of diseases [31–33]. Generally, miRNAs play multiple roles in tumor development, and studies have shown that miR-21, miR-142, miR-200a, miR-101, let-7c, miR-378e, miR-484, and miR-21-5p are closely associated with the development of LUAD. Although numerous studies have been conducted on the pathogenesis of LUAD, in-depth details have not been reported. To gain a deeper insight into the mechanism of LUAD and explore new therapeutic options, several studies have focused on miRNAs, which could be used as new diagnostic and prognostic biomarkers, and therapeutic targets for tumors.

The results of these studies have shown that differentially expressed miRNAs play multiple roles in tumor development, and interfering with their expression can suppress tumor cells *in vitro* and *in vivo*. We hypothesized that since miR-30b-3p has been shown to be differentially lowly expressed in ovarian cancer, hepatocellular carcinoma and glioma miR-30b-3p could be used as a marker for cancer diagnosis or a target for cancer therapy. In this study, qRT-PCR analysis showed that the expression of miR-30b-3p in LUAD tissues was significantly lower than that in normal tissues. Additionally, miR-30b-3p expression in the LUAD cell lines was significantly lower than that in human bronchial epithelial cells. Moreover, the OS was worse in patients with lower miR-30b-3p expression. Thus, miR-30b-3p appears to be oncogenic in the development of LUAD.

The next step is to examine whether miR-30b-3p's has an effect on LUAD. In order to enhance the regulatory effects of endogenous miRNAs for function acquisition, miRNA mimics and Agomirs were synthetically synthesized using chemical methods. We achieved overexpression of miR-30b-3p in LUAD cell lines by transfection of miR-30b-3p mimics or Agomirs into A549 and H1975 cells. In the development of multicellular organisms, the regulation of cell proliferation is central to tissue morphogenesis, and uncontrolled cell proliferation is also critical to the pathology of diseases, such as cancer [34]. Our results showed that induced expression of miR-30b-3p inhibited the proliferation and invasion of A549 and H1975 cells *in vitro* and the growth of implanted tumors in nude mice *in vivo*. Under normal physiological conditions, the cells undergo proliferation and apoptosis at a slow rate to maintain a constant cell population, and apoptosis is a complex process of cell renewal involving both normal and pathological processes in the body [35]. The results of the flow cytometric analysis show that overexpression of miR-30b-3p promotes apoptosis. In cells, dysregulation of cell cycle mechanisms and distribution of components reflect the importance of an aberrant cell cycle for the proliferative phenotype. Therefore, there is a need to study cell proliferation and determine the distribution of cells in each phase of the cell cycle. Since the decision to proliferate is made during the G1 phase when DNA synthesis begins and before the rest of the cell cycle proceeds, measuring DNA synthesis at this stage can reveal the state of growth regulation in cell culture [36]. We examined the cell cycle using flow cytometry and showed that G1 phase increased, S phase decreased, and G2/M phase changed insignificantly after induction of miR-30b-3p expression compared to the NC group.

A miRNA performs its biological function by regulating the expression of a target gene [37]. We predicted that *METTL7B* is a potential target gene for miR-30b-3p by using an online target gene prediction website, based on the prediction of the binding of the miRNA seed region to the mRNA molecule. The seed region refers to the most conserved fragment of the miRNA, which is usually complementary to the target site on the mRNA 3′-UTR [38]. We further confirmed that *METTL7B* is the direct target gene of miR-30b-3p using a dual luciferase gene reporter assay, qRT-PCR, and Western blot analysis. We also found a strong negative correlation between METTL7B and miR-30b-3p expression, thus providing indirect evidence that *METTL7B* is a downstream direct target gene of miR-30b-3p.

On this basis, we further performed rescue experiments, in which METTL7B overexpression was able to significantly reverse the effects of miR-30b-3p in miR-30b-3p overexpressing cells. Specifically, we used miRNA mimics and gene overexpression technologies to simultaneously overexpress miR-30b-3p and *METTL7B* to determine whether the effects of miR-30b-3p upregulation alone on the biological function of LUAD cells could be restored. We found that the proliferation and invasion ability of cells inhibited by miR-30b-3p were restored by upregulating METTL7B expression in LUAD cells. Additionally, we further examined the apoptosis and cell cycle distribution of LUAD cells and found that upregulation of METTL7B expression was able to partially reverse the miR-30b-3p-induced alteration of apoptosis and cell cycle distribution of LUAD cells. These results suggest that miR-30b-3p may be involved in the development of LUAD by directly targeting *METTL7B* to inhibit cell proliferation and invasion, promote apoptosis and arrest LUAD cells in the G0/G1 phase. There are some shortcomings in this study, including the following: (1) one miRNA can target multiple target genes, and this study only focuses on the regulatory effect of miR-30b-3p on METTL7B, so the mechanism by which miR-30b-3p acts may not be fully understood. (2) More clinical samples are needed to measure the expression of miR-30b-3p. (3) The downstream regulatory network of the miR-30b-3p/METTL7B axis has not been explored.

## Data Availability

No data were used to support this study.
